# Host-pathogen interactions of clinical *S. aureus* isolates to induce infective endocarditis

**DOI:** 10.1080/21505594.2021.1960107

**Published:** 2021-09-07

**Authors:** Christian Schwarz, Yasemin Töre, Vanessa Hoesker, Sabine Ameling, Katja Grün, Uwe Völker, P. Christian Schulze, Marcus Franz, Cornelius Faber, Frieder Schaumburg, Silke Niemann, Verena Hoerr

**Affiliations:** a Translational Research Imaging Center, Clinic for Radiology, University Hospital Muenster, Muenster, Germany; b Interfaculty Institute for Genetics and Functional Genomics, University Medicine Greifswald, Greifswald, Germany; c DZHK (German Centre for Cardiovascular Research), Partner Site Greifswald, Greifswald, Germany; d Department of Internal Medicine I, Jena University Hospital, Jena, Germany; e Institute of Medical Microbiology, University Hospital Muenster, Muenster, Germany; f Institute of Medical Microbiology, Jena University Hospital - Friedrich Schiller University Jena, Jena, Germany; g Medical Physics Group, Institute of Diagnostic and Interventional Radiology, Jena University Hospital - Friedrich Schiller University Jena, Jena, Germany

**Keywords:** endocarditis, clinical isolates, MRI, *Staphylococcus aureus*, pathomechanisms

## Abstract

To evaluate potential pathomechanisms in the induction of infective endocarditis (IE), 34 *Staphylococcus aureus* (*S. aureus*) isolates, collected from patients with *S. aureus* endocarditis and from healthy individuals were investigated both *in vitro* and *in vivo. S. aureus* isolates were tested *in vitro* for their cytotoxicity, invasion and the association with platelets. Virulence factor expression profiles and cellular response were additionally investigated and tested for correlation with the ability of *S. aureus* to induce vegetations on the aortic valves *in vivo*. In an animal model of IE valvular conspicuity was assessed by *in vivo* magnetic resonance imaging at 9.4 T, histology and enrichment gene expression analysis. All *S. aureus* isolates tested *in vivo* caused a reliable infection and inflammation of the aortic valves, but could not be differentiated and categorized according to the measured *in vitro* virulence profiles and cytotoxicity. Results from *in vitro* assays did not correlate with the severity of IE. However, the isolates differed substantially in the activation and inhibition of pathways connected to the extracellular matrix and inflammatory response. Thus, comprehensive approaches of host-pathogen interactions and corresponding immune pathways are needed for the evaluation of the pathogenic capacity of bacteria. An improved understanding of the interaction between virulence factors and immune response in *S. aureus* infective endocarditis would offer novel possibilities for the development of therapeutic strategies and specific diagnostic imaging markers.

## Introduction

Infective endocarditis (IE) is a life-threatening disease and can be observed as acute and chronic infection of the endocardium, both valvular and parietal, and the valve itself. In many cases both starting point and duration of the infection are unknown and patients present with signs of acute illness. The Gram-positive bacterium *Staphylococcus aureus* (*S. aureus*) is one of the most frequent causative infectious agents [1]. IE is usually preceded by an abnormality in the heart valves due to congenital or degenerative diseases, resulting in mechanical alteration of the endothelium. Under these conditions, or in response to inflammation-associated endothelial dysfunction, the endothelial cells are activated and the subendothelial matrix is exposed in case of cellular damage. This results in a rapid accumulation of activated platelets, neutrophils and monocytes, and overexpression of fibrinogen, fibronectin and von Willebrand Factor (vWF) [2–4]. These factors provide favorable conditions for circulating *S. aureus* to attach directly or via a fibrin bridge with their numerous adhesins such as, for example, clumping factor A (ClfA), fibronectin-binding proteins (FnBPs) or protein A (SpA) [5,6]. Other adhesins such as the extracellular adherence protein (Eap) are able to activate more platelets [7,8] and bacterial toxins such as α-toxin and superantigens can attack different host cells, leading to further activation and destruction of both immune and endothelial cells [9,10]. The *S. aureus* virulence factors (see also supplementary table 1) are tightly regulated by global regulators including *agr, sarA, sae* or *sigB*. Presumably, different networks of multiple virulence genes are expressed in response to different host signals found in blood and specific target tissues, and various virulence factors are released during the infection process, and to evade host defense [11]. In the first reaction to an infection, the innate immune response is activated by pattern recognition molecules, which attract phagocytic cells such as neutrophils and macrophages to the site of infection. However, *S. aureus* has developed mechanisms to escape the immune system. These strategies can be mediated, e.g. by the ability of SpA to bind immunoglobulin G Fc fragments which impedes phagocytosis. Other mechanisms are frequently based on the expression of immune evasion proteins such as chemotaxis inhibitory proteins of *S. aureus* (CHIPS) and staphylococcal complement inhibitor (SCIN), which avoid the attack by the complement system [12]. In addition, the bacteria have the ability to hide from the immune system by invading host cells. This process is mainly promoted by *S. aureus* FnBPs and fibronectin. Internalization of *S. aureus* results in a cell defense response through expression and release of proinflammatory cytokines. Within the eukaryotic cells, the bacteria can either persist [13] or attack and destroy their cellular host from within [14], leading to further damage of the heart valve tissue [15]. The extent to which *S. aureus* acts as a pathogen is strongly regulated by the bacterial cell itself, its virulence profile and certain circumstances including the host environment, the factors of which are largely unknown [14,16].

To identify the key virulence factors for induction and progression of IE, several previous studies on pathomechanisms have been performed, using different knock-out mutants [17]. However, the downregulation of specific genes frequently has an impact on the expression of other virulence factors [18]. Therefore, to analyze virulence strategies in a comprehensive picture, *in vivo* investigations of clinical isolates are needed [19]. To this end, we established an *S. aureus* strain collection of 24 clinical endocarditis isolates from humans. We representatively characterized their manifestation in a mouse model of IE by MRI and determined their invasive, proinflammatory and cytotoxic features on a cellular and molecular level.

## Methods

### Bacterial isolates and culture

34 *S. aureus* isolates were collected either from patients diagnosed with IE (n = 24) or from the nasal swabs of healthy individuals (n = 10) at the University Hospital Muenster, Muenster, Germany. Isolates from IE were from blood cultures (n = 23) and heart valves (n = 1). All patient isolates were subjected to a sensitivity test using the VITEK 2 system (bioMérieux, Nuertingen, Germany). For the patient isolates, spa sequence typing was additionally conducted by amplification of the variable region of protein A by PCR followed by sequencing according to Harmsen et al. [20]. The assignment of the spa types was realized with the software Ridom StaphType (Ridom GmbH, Wuerzburg, Germany). All features are summarized in the supplementary table 2.

### Bacterial cultures

In preparation for the infection experiments bacteria from overnight culture in Tryptic Soy Broth (TSB) (shaking conditions, 37 °C) were adjusted to OD_578_ 0.1 in TSB. After 3 h of growth (shaking conditions, 37 °C) the bacteria were adjusted to OD_578_ 1 and stored in aliquots at −20 °C until use. From a previously frozen aliquot the number of colony forming units (CFU) was determined after serial dilutions of the bacterial suspensions on blood agar and overnight incubation at 37 °C.

### Cell culture

For invasion and cytotoxicity assays primary human umbilical vein endothelial cells (HUVEC) were used. Cells were isolated by collagenase treatment as described previously [21] and cultured in Endothelial Cell Growth Medium (PromoCell, Heidelberg, Germany) on fibronectin coated dishes. HUVECs were used from passage 1 or 2.

For analysis of the cellular response to *S. aureus* infection the endothelial cell line EA.hy926 (ATCC, CRL-2922) was used. The cells were cultured in Dulbecco’s modified Eagle medium (DMEM, Biochrom, Berlin, Germany), supplemented with 10 % fetal calf serum (FCS, Biochrom, Berlin, Germany) and 1xHAT supplement. Both cell types were seeded at 40,000 cells/cm^2^ in 12 well plates (Corning, Costar, tissue culture-treated surface, Wiesbaden, Germany) 2–3 days before the experiment and were used at 90–100 % confluence. On the day of the experiment cells of one well were detached with trypsin-EDTA and the cell number was determined using an automated cell counter (TC20, Bio-Rad, Feldkirchen, Germany). EA.hy926 cells were used until passage 60. Regular control experiments with well characterized *S. aureus* strains were used to check that cells from higher passages still showed the same behavior as cells from the lower ones [14].

### Flow cytometric invasion assay and cell death induction

Uptake of *S. aureus* into HUVECs was analyzed by flow cytometry as previously described [14]. In brief, a formalin (2 %)-fixed fluorescein-isothiocyanate-labeled bacterial suspension (OD_540_ 1) was prepared as described [22] and added to the cells (100 μl). Bacterial uptake at 3 h post infection was analyzed by flow cytometry. Data represent adherent and internalized bacteria, with a predominant proportion of internalized bacteria. To analyze cell death induction, cells (seeded 2 days before infection) were incubated with living bacteria at a MOI of 50 for 3 h, followed by a 30-minute lysostaphin treatment to lyse all extracellular staphylococci. Subsequently, fresh culture medium was added to the cells. 24 h post infection, cells were detached with trypsin/EDTA and stained with 10 μg/mL propidium iodide (PI). The PI staining of the cells was analyzed by flow cytometry [14].

### Bacteria-platelet-associates

All studies were performed with the blood donors giving informed consent and have been approved by the local ethics committee. Blood donors had not taken any medication affecting platelet function for at least 2 weeks before the study. Platelet-rich plasma (PRP) from trisodiumcitrate-anticoagulated blood was prepared by centrifugation at 250 x g for 10 min at room temperature.

Platelets were diluted with PBS to 2.5 × 10^4^ cells/µl and labeled with an anti-CD42a PE-conjugated antibody for 30 min. Labeled platelets in PRP were activated with 1 U/ml α-thrombin (control cells were not activated) for 3 min. To inhibit fibrin polymerization, experiments were performed in the presence of GPRP (1.25 mmol/L). Subsequently, platelets were incubated with fluorescent labeled *S. aureus* isolates (Syto13, 2 μmol/L, 10 min), at a MOI of 10 for 15 min, and conjugate formation was measured immediately by flow cytometry [5].

### RNA isolation and quantitative reverse transcription PCR (RT-qPCR) from cell culture

To analyze the expression of bacterial virulence factors and global regulators RT-qPCR was used. *S. aureus* bacteria were cultured for 3 h in TSB and RNA was extracted using RNeasy Mini kit (Qiagen GmbH, Hilden, Germany) followed by a purification step using RNeasy MinElute Cleanup kit (Qiagen GmbH, Hilden, Germany) according to manufacturer´s instructions und the suggestions of the protocol described by Garzoni et al. 2007 [23]. Concentration and purity of eluted RNA were analyzed by measuring the factors A260/280 and A260/230 using the NanoPhotometer P330 (Implen, Muenchen, Germany).

cDNA was obtained using the kit QuantiTect reverse transcription (Qiagen GmbH, Hilden, Germany). For RT-qPCR iQSYBR Green Supermix (Bio-Rad Laboratories GmbH, Duesseldorf, Germany) was used. The reaction mixtures were incubated for 15 min at 95 °C followed by 40 cycles of 15 s at 95 °C, 30 s at 55 °C and 30 s at 72 °C using the C1000 Thermal Cycler (Bio-Rad Laboratories GmbH, Duesseldorf, Germany). Each sample was analyzed in technical duplicates. Criteria for validation of the results were similarity of Cq values of technical replicates, purity of negative controls and correctness of melt curves. PCR efficiencies, melting-curve analysis and expression rates were calculated with the Bio-Rad CFX Manager Software. To analyze the expression of the virulence factors of *S. aureus*, the gene for gyraseB (*gyrB*) was taken as house keeping gene. All data were normalized to this gene. Utilized primers are listed in supplementary table 3.

### PCR detection of Staphylococcal enterotoxins

*S. aureus* isolates (endocarditis isolates and the reference strains ATCC 13565 – *sea* expressing *S. aureus* strain, ATCC 14458 – *seb* expressing *S. aureus* strain, ATCC 19095 – *sec* expressing *S. aureus* strain and KN813 – *tst S. aureus* expressing strain [24]) were grown on blood agar at 37 °C overnight. Subsequently, several colonies were harvested and resuspended in Tris-EDTA buffer containing 200 μg/mL lysostaphin (Sigma-Aldrich Co. LLC, Muenchen, Germany). After cell lysis, genomic DNA was extracted using QIAamp DNA Minikit (Qiagen GmbH, Hilden, Germany) according to manufacturer’s instructions, and DNA concentration was determined by NanoPhotometer P330 (Implen GmbH, Muenchen, Germany). Primers are described in supplementary table 3. PCR amplifications were performed using thermal cycler (ICycler, Bio-Rad Laboratories GmbH, Duesseldorf, Germany), with the following thermal cycling profile: initial denaturation step at 94 °C for 5 min, followed by 35 cycles of 2 min at 95 °C, 2 min annealing at 50 °C (*sea* and *tst*) or 57 °C (*seb* and *sec*) and 1 min extension at 72 °C, and final extension at 72 °C for 7 min [25,26]. Genomic DNA was added to PCR mix containing 1.5 mM MgCl_2_, 0.2 mM of each deoxynucleoside triphosphates, 2 pM each of forward and reverse primers and Taq polymerase (Segenetic, Borken, Germany). PCR products were analyzed by 2 % agarose gel electrophoresis and GelRed (Sigma-Aldrich Co. LLC, Muenchen, Germany) staining.

### *Determination of host cell response to* S. aureus *infection*

Response of EA.hy926 to *S. aureus* invasion was determined 8 h post infection by RT-qPCR. Infection was performed as described for intracellular cytotoxicity. At 8 h post infection, cells were detached with EDTA-trypsin, centrifuged, suspended in RNAprotect (Qiagen GmbH, Hilden, Germany). After RNA extraction (RNeasy Mini kit and RNeasy MinElute Cleanup kit, Qiagen GmbH, Hilden, Germany) cDNA was synthetized (Quantitect reverse transcription kit, Qiagen GmbH, Hilden, Germany) following the manufacturer’s recommendations.

Real-time amplification was done with the iQ SYBR Green Supermix (Bio-Rad Laboratories GmbH, Duesseldorf, Germany) and specific primers (supplementary table 3) on an iCycler iQ real-time PCR-system (Bio-Rad Laboratories GmbH, Duesseldorf, Germany). The reaction mixtures were incubated for 15 min at 95 °C followed by 40 amplification cycles (15 s at 95 °C, 30 s at 55 °C, 30 s at 72 °C). Each sample was analyzed in technical replicates. Bio-Rad CFX Manager Software was used to calculate PCR efficiencies, melting-curve analysis, and expression rates. α-actin and GAPDH were used as endogenous controls to normalize expression levels. Data are presented as normalized fold change in expression compared to controls (non-infected cells) using the ΔΔCt method.

### *Host cell response to* S. aureus *infection* in vivo

To investigate the gene expression profile of the five experimental study groups (naïve controls, sham-operated mice and mice infected with *S. aureus* isolates 17, 30 or 33 in the IE model), RNA was extracted from heart tissue using peqGOLD TriFast reagent (VWR, Darmstadt, Germany) according to the manufacturer’s instruction after tissue maceration in liquid nitrogen. Concentration of extracted RNA was determined using a spectrophotometer (NanoDrop ND-1000, Peqlab Biotechnologie GmbH, Erlangen, Germany). For cDNA synthesis, total RNA of every study group (naïve controls – n = 8, sham-operated mice – n = 4 and mice infected with *S. aureus* isolates 17 – n = 3, 30 – n = 4 or 33 – n = 3 in the IE model, 3 to 8 mice each) was pooled to obtain 500 ng of total RNA. Reaction was performed using RT^2^ First Strand Kit (SABiosciences, USA) according to the manufacturer’s instructions. Afterward, cDNA concentrations of each sample were measured using a spectrophotometer (Nanodrop ND-1000, Peqlab Biotechnologie GmbH, Erlangen, Germany) and stored at −20 °C until further use.

For gene expression profiling, RT^2^ Profiler PCR Array Inflammatory Response & Autoimmunity as well as Extracellular Matrix & Adhesion Molecules (SABiosciences, USA) was conducted according to the manufacturer’s instructions. 102 µl of cDNA was added to the experimental cocktail containing 1350 µl of 2x RT^2^ SYBR-Green ROX FAST Mastermix (SABiosciences, USA) and 1248 µl RNAse-free water. 25 µl of this cocktail were added to each well of the 96-well RT^2^ Profiler PCR Array plate (SABiosciences, USA) and wells were capped using Haetin-Sealing Film (SABiosciences, USA). The plate was placed in the Cycler “Step One plus” Real Time PCR System (Applied Biosystem/Thermo Fisher Scientific, Germany). The cycler was used for real-time PCR with a two-step cycling program with 1 cycle of 95 °C for 10 min (activation of HotStart DNA polymerase) followed by 40 cycles of 95 °C for 15 s and 60 °C for 60 s (detection and recording of SYBR® Green fluorescence from every well during the annealing step of every cycle). Finally, for quality control, a melting curve was generated.

Data analysis was performed using Ct values (cycle threshold). The Ct was calculated for each well using the step one software v2.3 (Applied Biosystem/Thermo Fisher Scientific, Germany). For quality control, Genomic DNA Control, Reverse Transcription Control and Positive PCR Control were used according to the instructions included in the kit. A ΔCT value for each gene in each plate was then calculated using a mean value of five housekeeping genes for normalization.

### *Mouse model of* S. aureus*-induced IE*

Female C57BL/6 mice with an average body weight of 19.4 ± 1.3 g and an age of 8–12 weeks were used in this study. For the induction of *S. aureus* IE, a surgical intervention was performed, and a 32-G polyurethane catheter (13 mm of the tube were cut and heat-sealed at both ends) was placed via the right carotid artery at the aortic root to induce trauma and endothelial damage on the aortic valve. The aortic valves were reliably reached by slowly advancing the catheter until vibration occurred [27,28]. During surgery, the animals were anesthetized with 2 % isoflurane. Analgesics (Carprofen; Rimadyl (5 mg/kg body weight, Pfizer Animal Health, NY, USA)) were applied pre- and postoperation. An inoculation with the three *S. aureus* isolates 17, 30 and 33 was conducted 24 h post catheter placement by intravenous (i. v.) injection of 10^5^ CFU in 100 µl through the tail vein. A sham-operated group of animals without infection received 100 µl of phosphate-buffered saline (PBS) 24 h after surgical intervention. 48–56 h post catheter placement, the animals were sacrificed by transcardial perfusion and organs and catheter were harvested for molecular and microbiological analysis as well as histopathology.

### Clinical score

All animals were examined and screened daily with respect to body weight, body temperature, respiration, physical appearance, behavior, and wound healing from surgical interventions. Each day, score points were given for each health feature and were added up as clinical score, representing a quantitative measure of the severity of the disease [28].

### MR imaging

Cardiac MRI was performed under isoflurane anesthesia at 9.4 T on a BioSpec 94/20 small animal MRI system equipped with a 1 T/m gradient system. Cine-images of 20 cardiac frames were acquired using a mouse body quadrature volume coil with an inner diameter of 35 mm (Rapid Biomedical, Rimpar, Germany) and ParaVision software version 5.1 (Bruker BioSpin, Ettlingen, Germany). During MRI, the animals were anesthetized with isoflurane (1.5–2.5 % isoflurane and 0.7/0.3 air/O_2_ mixture), and the physiological parameters of core body temperature and respiration were monitored using an MRI compatible monitoring system (SA Instruments, Stony Brook, NY, USA). For imaging of the infected aortic valves, a self-gated CINE-UTE sequence was used (TR/TE: 5/0.31 ms, FA: 15°, FOV: (3.20 cm)^2^, MTX: 256 × 256, section: 1 mm, scan duration: 12:08 min) and 20 images per cardiac cycle were reconstructed retrospectively.

### Microbiological analysis

CFU counts of spleen, lung, kidney, liver, myocardium, aortic arch, aortic valves and catheter were determined as previously described [28].

### Histology

#### Gram- and HE-staining

Serial cryosections of heart valves were prepared with a thickness of 5 µm for histological analysis. *S. aureus* was Gram-stained according to standard protocols. Hematoxylin and eosin staining (HE) were used to detect basophilic components (DNA/RNA) and eosinophilic structures (collagen/muscle).

#### Immunofluorescence labeling of pan-leukocytes (CD45) and macrophages (F4/80)

For immunofluorescence labeling, 5 µm thick cryosections from heart valve tissue of all experimental groups were fixed in ice-cold acetone for 10 min followed by ice-cold 80 % methanol for 5 min. The rat-anti-mouse monoclonal antibodies of 30-F11 (BD Biosciences, Heidelberg, Germany) and FA-11 (Bio-Rad Laboratories GmbH, Muenchen, Germany) were applied for CD45 and F4/80 detection respectively, and allowed to incubate for 60 min at room temperature. The cryosections incubated with the rat-anti-mouse monoclonal antibodies were rinsed three times in TBS-T washing buffer and were then incubated with Cy3-conjugated AffiniPure Donkey Anti-Rat IgG (Jackson Immunoresearch Laboratories Inc., Pennsylvania, USA) for 45 min at room temperature. After rinsing in TBS-T buffer and distilled water, sections were mounted in Vectashield H1200 mounting medium containing DAPI (Linaris biologische Produkte GmbH, Wertheim-Bettingen, Germany) and stored at −20 °C. Antibody specificity control staining was performed in accordance but by omitting the primary antibodies. Immunofluorescence labeling was analyzed with the LSM 9000 (Aiyscan 2) microscope using the ZEN software (both Carl Zeiss, Germany).

### Statistical analysis

#### Group analysis

Statistical analyses were performed with the software GraphPad software version 5 (GraphPad Software, La Jolla California, USA). Bacterial loads as well as gene expression profiles were analyzed statistically using One-way-ANOVA followed by Bonferroni-posttest. A value of p < 0.05 was considered as significant.

#### Principal component analysis

Principal component analysis (PCA) was performed using SPSS software (IBM Corp. Released 2020. IBM SPSS Statistics for Windows, Version 27.0. Armonk, NY: IBM Corp, USA). The analysis was based on the following three principal components (PC): first PC = *S. aureus*-platelet-associates, second PC = cytotoxicity, third PC = invasion [29].

#### Enrichment statistical analysis

The enrichment analysis was performed with Ingenuity Pathway Analysis software (IPA, content version 52,912,811) and results were visualized using R (http://www.R-project.org, R version 4.0.2 (06/2020), “Taking Off Again”). The one-sided Fisher-Exact test was performed for 3 clinical isolates and control (sham-operated mice) based on gene expression data of selected panels (fold change filter FC > |2|, PAMM-013ZC-12-RT2 Profiler PCR Array Mouse Extracellular Matrix and Adhesion Molecules, PAMM-077ZC-12-RT2 Profiler PCR Array Mouse Inflammatory Response and Autoimmunity, Qiagen GmbH, Hilden, Germany). Negative log_10_(*p*-value) of most enriched (n = 3 per group) categories as well as z-scores (indicating predicted activation or inhibition of pathways) were comparatively depicted for categories (n = 10) of diseases and biofunctions for all clinical isolates.

### Ethics approval

All animal experiments were approved by the North Rhine-Westphalia Agency for Nature, Environment, and Consumer Protection (Landesamt für Natur, Umwelt und Verbraucherschutz Nordrhein-Westfalen-LANUV; ID 87–51.04.2011.A003; ID 84–02.04.2015.A581).

The isolation of human cells as well as the collection of bacterial isolates was approved by the local ethics committee (Ethik-Kommission der Ärztekammer Westfalen-Lippe und der Medizinischen Wilhelms-Universität Münster, Az. 2018–743-f-S). All methods were performed in accordance with the relevant guidelines and regulations. This study conforms to the principles outlined in the Declaration of Helsinki.

### Availability of data and material

All datasets generated for this study are included in the article, Supplementary Material and a DOI. All datasets generated for this study can be accessed here: 10.6084/m9.figshare.14974257.

## Results

### S. aureus *isolate collection*

**Figure 1.**
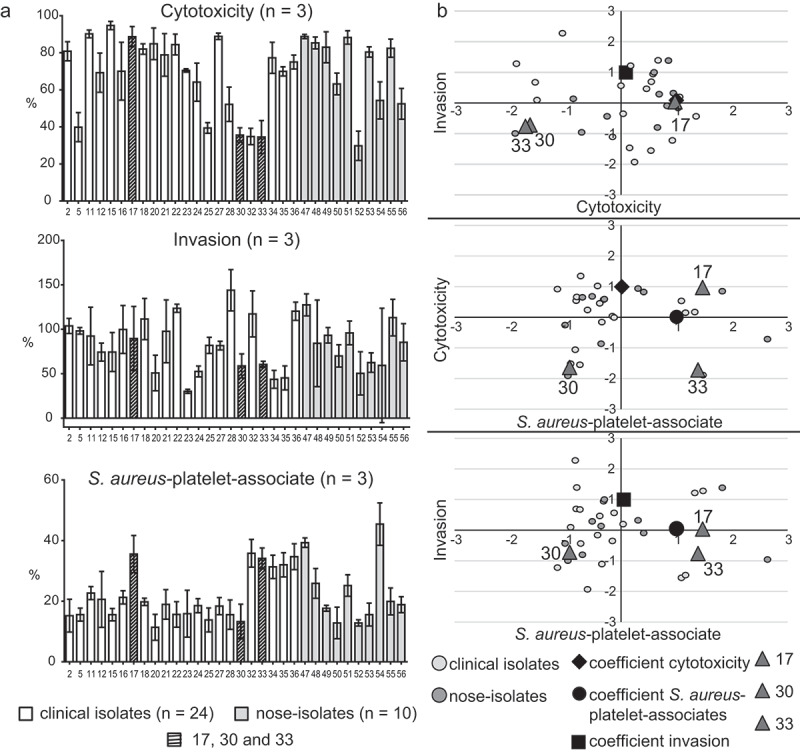
Cytotoxic properties, invasiveness and the ability to form *S. aureus*-platelet-associates as mean ± SD of a *S. aureus* isolate collection (34 isolates) isolated from patients with approved IE (white, *n* = 24) as well as from nasal swabs of healthy individuals (gray, *n* = 10) (a). Principal component analysis (PCA) of pathogenic profiles using cytotoxicity, invasion and platelet-associates as principal components (PCs), explaining 100 % of the total variation. The analysis shows that the isolates of the two different collection sites (*S. aureus* IE and nasal swabs of healthy individuals) give comparable variation along the three PCs. Isolate 17, 30 and 33 differed most in their pathogenic profiles

34 *S. aureus* isolates were collected from patients with *S. aureus* endocarditis (n = 24) and from nasal swabs of healthy individuals (n = 10). *In vitro* assays of cytotoxicity, invasion and *S. aureus*-platelet association showed diverse behavior over all isolates from the two different origins (Figure 1a). The results were analyzed by PCA using the variables of *S. aureus*-platelet association (PC 1), cytotoxicity (PC 2) and invasion (PC 3) as the three main principal components explaining 100 % of the variation. The analysis did not show clusters based on the isolate collection site (Figure 1b). Three *S. aureus* isolates, isolate 17 (PC 1 = 1.47, PC 2 = 0.97, PC 3 = 0.04), 30 (PC 1 = −0.93, PC 2 = −1.64, PC 3 = −0.72) and 33 (PC 1 = 1.38, PC 2 = −1.72, PC 3 = −0.77) showing substantially different scores were selected for further *in vitro* and *in vivo* analysis.

### *Virulence profiles of* S. aureus *isolates 17, 30 and 33* in vitro

**Figure 2.**
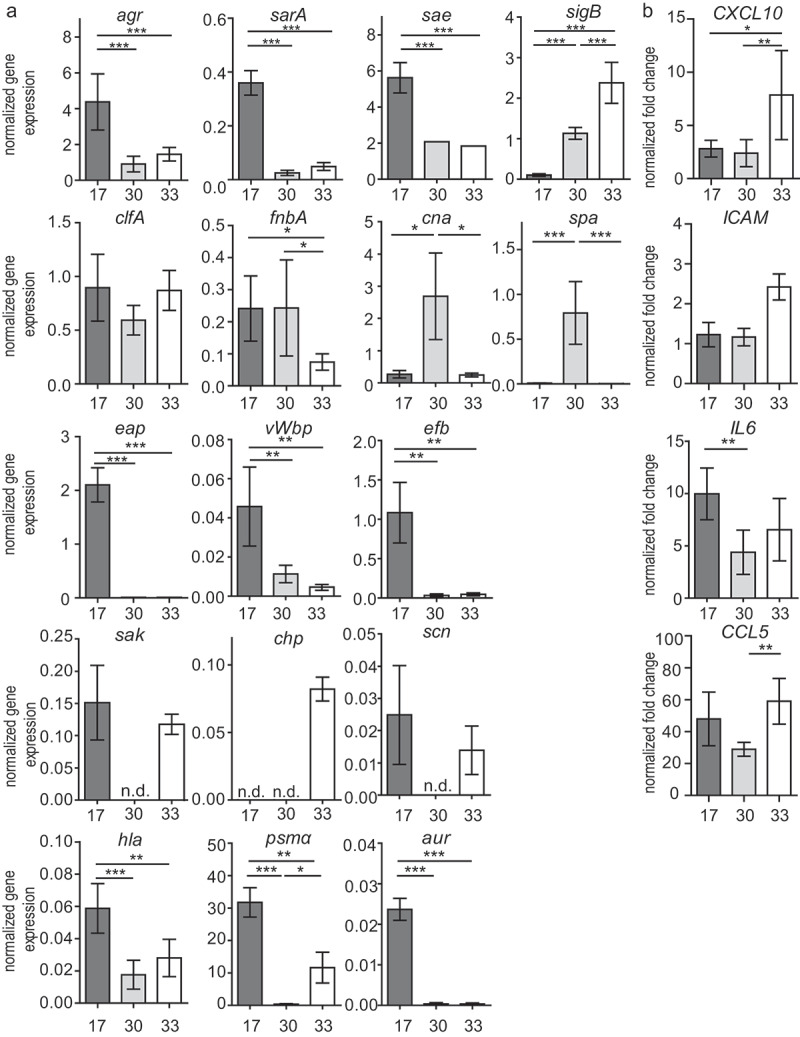
Gene expression patterns (RT-qPCR) of major virulence factors in the three selected isolates (17, 30, 33) 3 h post inoculation: general regulatory proteins, adhesion proteins (MSCRAMMs and SERAMs), immune evasion proteins, toxins, and proteases (a). Gene expression patterns (RT-qPCR) of major immunomodulatory (CCL5, CXCL10 and IL6) and proangiogenic (ICAM) factors in EA.hy926 cells induced by the three selected isolates (17, 30, 33) 8 h post infection (b). The results are displayed as bar charts representing the mean ± SD. *p < 0.05, **p < 0.01, ***p < 0.001, One-way-ANOVA with Bonferroni posttest; n.d. not detected

Compared to isolates 30 and 33, isolate 17 showed substantially higher cytotoxicity and invasion values and additionally induced pronounced formation of *S. aureus*-platelet-associates (Figure 1a). Isolate 30 showed low characteristics in all three features, while isolate 33 exhibited low cytotoxicity and invasion but high levels of *S. aureus*-platelet-associates. These results were also supported by the isolate-specific expression profiles of virulence factors. Expression levels of major virulence factors (summarized and described in supplementary table 1) showed significant differences between the three selected isolates (Figure 2a). Especially isolate 17 expressed high levels of *agr, sarA, sae*, and of the adhesion-proteins *clfA, fnbA, eap, efb* and *vWbp*, accompanied by high levels of *hla, psmα, sak* and *aur*. In isolate 30, the expression level of *sigB* was increased compared to isolate 17. The expression levels of *spa* and *cna* were higher compared to isolates 17 and 33. Isolate 33 showed the highest expression of *sigB* and *chp*, whereas the genes for SCIN, SAK and PSMα were expressed similarly to isolate 17. Genes for the staphylococcal enterotoxins Sea, Seb and for TSST-1 were not detected in any of the three isolates by PCR (supplementary figure 2). In isolate 33, *sec* was present.

The host cells (EA.hy926) responded to the infection with an increased gene expression of *CCL5, ICAM-1, IL-6*, and *CXCL10*. Strikingly, the overall response to isolate 30 was lower compared to the other two. Host cells expressed significantly higher levels of *ICAM-1* as a reaction to isolate 33 in comparison to both isolate 17 and 30 (Figure 2b).

### In vivo *characterization of the* S. aureus *isolates 17, 30 and 33*

**Figure 3.**
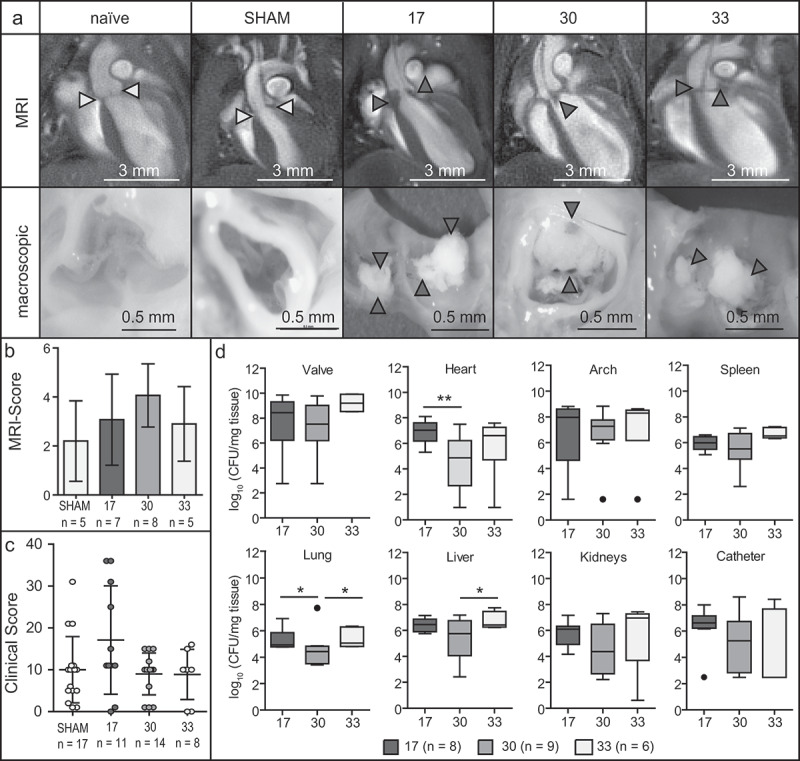
Representative MR (upper row) and macroscopic (lower row) images (a) of the five experimental groups (naïve controls (*n* = 5) and sham-operated mice (*n* = 5) and mice infected with *S. aureus* isolates 17 (*n* = 7), 30 (*n* = 8) or 33 (*n* = 5) in the IE model) 24 h post the induction of IE. MRI score (b) and clinical score (c) of the three *S. aureus* endocarditis infections (17, *n* = 11; 30, *n* = 14 or 33, *n* = 8) and the sham-operated group (*n* = 17) represented as mean ± SD. CFU counts of different organs including the aortic valves (d) for IE infections induced by the bacterial isolates 17 (*n* = 8), 30 (*n* = 9) and 33 (*n* = 6). The results are displayed as box and whiskers plots representing data between the first and third quartiles with the band standing for the second quartile (=median). Whiskers represent lowest and highest data within 1.5 interquartile ranges of the lower and upper quartile. *p < 0.05, **p < 0.01, Mann–Whitney-U-test

The ability to induce endocarditis was assessed for the three different *S. aureus* isolates *in vivo* by MRI using a well characterized and established mouse model of IE. MR images revealed pronounced valve thickening, hypointensities and pendulum-like masses on the valves after inoculation with each of the three *S. aureus* isolates 24 h post infection. Valves of healthy mice or mice having received sham surgery did not show such conspicuities (Figure 3a). Corresponding MRI scores revealed substantially elevated values for each of the three infection groups (Figure 3b). Diagnostic findings were confirmed by macroscopic assessment, showing fulminant bacterial vegetations (Figure 3a). Overall disease symptoms and severity were quantified using clinical scores, and were correlated to the corresponding bacterial burden (in CFU/mg). The sham-operated group that had not received any infection showed mild to moderate clinical scores in the range of 4 to 20 (Figure 3c). In all three infection groups, nearly the same clinical scores were observed, and overall bacterial loads of 10^5^ to 10^7^ CFU/mg tissue were found in spleen, lung, liver and kidney. Higher bacterial counts were found on the aortic valves being highest (10^9^ CFU/mg tissue) and significantly elevated for infections with isolate 33 (Figure 3d, supplementary figure 1a), which was accompanied by the highest mortality (supplementary figure 1b).

### *Histopathological description of IE using* S. aureus *isolates 17, 30 and 33*

**Figure 4.**
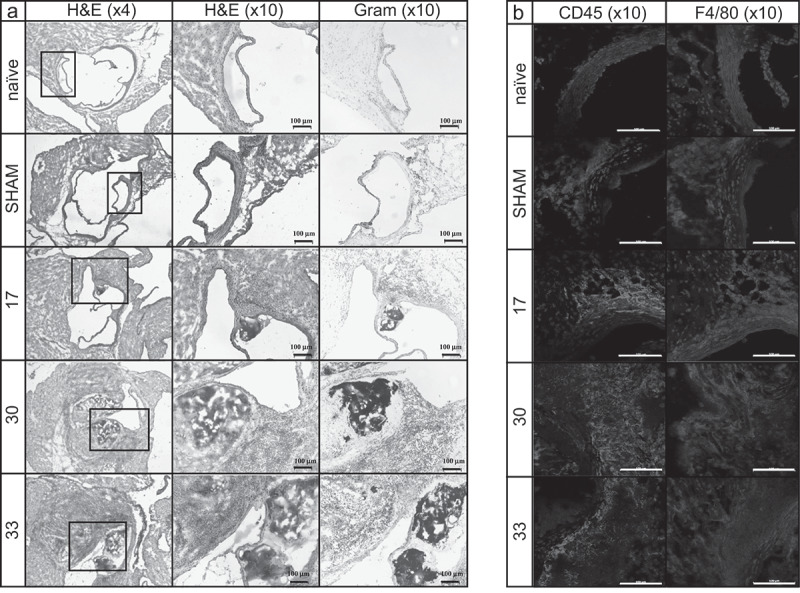
Histopathological analysis of aortic valves in comparison to the five experimental groups 24 h post induction of IE: naïve controls, sham-operated mice and mice infected with *S. aureus* isolates 17, 30 or 33 in the IE model. Hematoxylin-eosin staining (a; left column: ×4 magnification; middle column: ×10 magnification) and Gram staining (a; right column: ×10 magnification) showing massive immune cell infiltration. Immunofluorescence staining using CD45 as pan-leukocyte marker (b, red fluorescence, left column: ×10 magnification) and F4/80 as mouse macrophage marker (b, red fluorescence, right column: ×10 magnification) in representative tissue samples of the aortic valve. Blue fluorescence represents DAPI nucleic acid staining. The scale bars in the images illustrate 100 µm

To examine morphological abnormalities of the heart valves such as thickening or additional structures, histological analysis was performed on valve sections after infection with isolates 17, 30 and 33 (Table 1). Isolate-specific characteristic morphological and immunological alterations were observed mainly in the basal region and the valve-surrounding tissue of *S. aureus* infected animals. Most prominent bacterial vegetations, severe tissue destruction, infiltration of immune cells and pronounced fibrinous exudates were observed after infections with isolate 33 (Figure 4a). However, immunofluorescence labeling of CD45 as pan-leukocyte marker and F4/80 as mouse macrophage marker (red fluorescence) showed only mild-to-moderate invasion of leukocytes and only a very minor occasional presence of macrophages in the infiltrates (Figure 4b).

**Table 1.** Description of histological findings of Figure 4

	**HE and Gram**					**Immunohistology**	
	valve destruction	leaflet thickening	Gram-positive bacteria	fibrinous exsudates	infiltration of inflammatory cells	leukocyte infiltration	macrophage infiltration
naïve	0	0	0	0	0	0	0
SHAM	+	+	0	0	+	+	+
17	++	0	++	0	+/++	++	++
30	0	+++	++	++	++	++	++
33	+++	0	+++	+++	+++	+/++	+/++

### *Gen-array analysis of valve tissue infected with* S. aureus *isolates 17, 30 or 33*

**Figure 5.**
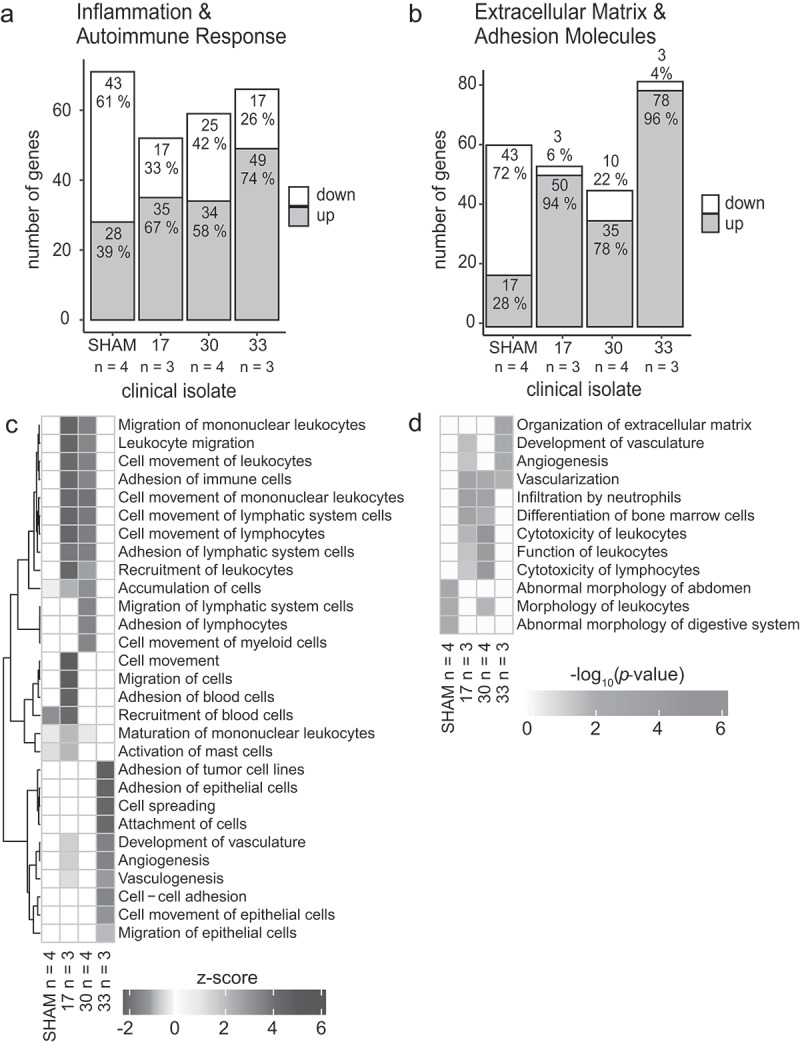
Gene expression in the aortic valves of the IE mouse model 24 h post infection with three clinical isolates of *S. aureus* (isolate 17 – *n* = 3, 30 – *n* = 4 and 33 –*n* = 3). A panel of genes associated with inflammation and autoimmune response (a) as well as extracellular matrix and adhesion molecules (b) are altered in non-infected control mice (sham– *n* = 4) and mice after infection with *S. aureus* clinical isolates 17, 30 and 33 (FC ≥|2|, one-sided Fisher-Exact test). Ingenuity pathway z score analysis of cellular and molecular biological functions (c) predicted increased activity of leukocyte migration in mice after infection with clinical isolate 17 and 30. Angiogenesis was predicted to be more pronounced in mice after infection with clinical isolate 33. -Log_10_(*p*-value) was given for the top three biological functions (d). Remodeling processes were more pronounced in mice after infection with isolate 33 compared to the other two

To identify potential pathways involved in the development of IE when different *S. aureus* isolates act as infectious agent, a panel of genes was investigated. This consisted of genes associated with inflammation and autoimmune response (Figures 5a, 82 genes) as well as genes of extracellular matrix and adhesion molecules (Figures 5b, 84 genes). Both, in sham-operated animals and in mice with IE, a high number of genes associated with the two pathways showed substantial alterations (Figure 5a,b). Tissue with sterile inflammation showed an upregulation of 28 % and 39 % of the genes, respectively. Pronounced upregulation was observed in tissue infected with isolate 17 (67 % and 94 %) and isolate 30 (58 % and 78 %), and most strongly upregulated gene expressions were identified in infections with isolate 33 (74 % and 96 %). Using ingenuity pathway analysis z-scores (Figure 5c) of cellular and molecular biological functions were predicted based on the two array datasets. These z-scores indicated increased activity of leukocyte migration in mice after infection with clinical isolates 17 and 30. In contrast, angiogenesis was calculated to be more prominent after infection with clinical isolate 33, as compared to the other two infections. Corresponding p-values of the three major biological functions (Figure 5d) demonstrated that remodeling processes accompanied by vascularization and angiogenesis were more pronounced in this infection group.

## Discussion

### In vitro *characterization of virulence factor expression and corresponding host cell interaction*

Over the last years several studies demonstrated that the phenotypes of *S. aureus* strains inducing IE and bacteremia were rather similar than distinctive. It could be shown that even under clearly defined disease manifestations, *S. aureus* isolates of IE and bacteremia revealed only subtle differences in their virulence profiles regarding adhesins, superantigens, toxins, etc. [16,30–33]. In our study, we pursued a different approach by first analyzing the pathogenicity of *S. aureus* isolates from endocarditis patients and commensal nose strains *in vitro*, followed by a detailed investigation of three selected strains *in vivo* that exhibited the most distinct virulence pattern *in vitro*.

The ability of *S. aureus* isolates to induce important steps in the pathogenesis of endocarditis was performed *in vitro* by assessing the bacterial adhesion to host cells and their invasion, as well as the subsequent host cell activation and destruction. These analyses showed that both commensally occurring and pathogenic bacteria displayed the same range of invasive and cytotoxic behavior, and also induced *S. aureus-*platelet-associates to the same extent. As a large variance between the different isolates was detected in these assays, three isolates with clearly different properties were characterized in more detail *in vitro* and *in vivo*. For isolate 17 overall high gene expression levels were observed for the major virulence regulatory system (*agr, sae*, and *sarA*), for some adhesion proteins (*fnbA, eap, efb, vWbp, clfA*), as well as for other virulence factors (*hla, psmα, sak*). The high gene expression for Hla corresponded to a high cytotoxicity and to the high expression of the global regulators mentioned above, which regulate *S. aureus* toxin production [34]. The observed high capability of host cell invasion of this isolate *in vitro* can be attributed to the expression of FnBPA [35] and Eap [31,36,37]. ClfA is known to facilitate *S. aureus* binding to fibrinogen/fibrin and leads to agglutination of cells [38]. It has also been shown to play an important role in the binding of *S. aureus* to activated platelets [5,39]. Thus, the high expression of *clfA* correlates well with the high rate of platelet-associates.

Isolate 30 and 33 showed similar and rather low levels of *efb* and *vWbp* as well as low toxin production (low gene expression of *agr* and *hla, psmα, aur*), which was reflected in low cytotoxicity. While especially isolate 33 showed low *fnbA* expression, both isolates displayed a low expression of *eap* which might be the reason for the observed reduced host cell invasion [40]. Isolate 33 formed more bacteria-platelet-associates than isolate 30, possibly due to higher *clfA* expression. After infection of host cells, all three isolates led to a different host cell response. Of note, although isolates 30 and 33 showed similar characteristics in invasion and cytotoxicity, isolate 30 resulted in the lowest host cell response to infection. To test whether conclusions can be drawn from *in vitro* assay for the course of IE *in vivo*, the three characterized isolates were also investigated in an endocarditis mouse model. Frequently, patterns of virulence profiles differ strongly between *in vivo* and *in vitro* conditions [34], as well as between different organ tissues [18]. Since *S. aureus* has evolved a regulatory network to control the expression of virulence factors, it is able to survive and adapt to different environmental niches. In addition, selectively induced mutations of virulence factor genes might be of distinct importance in different niches [41]. Strongly isolate-associated manifestations of IE *in vivo* were recently described by Liesenborghs et al. [2]. In a mouse model of IE the authors of that study investigated the bacterial colonization of different *S. aureus* clinical isolates and knockout mutants, and identified Sortase A-dependent adhesins such as vWbp and ClfA as most relevant for the initial adhesion of *S. aureus* on the endothelium of the heart valves. Other studies observed the additional involvement of SarA and Agr [17].

### Correlation between pathohistological findings

Our results showed that all three *S. aureus* isolates resulted in reliable infections of the aortic valves *in vivo*, accompanied by visible abnormalities and high bacterial loads on the valves, and severe disease symptoms. However, *S. aureus* isolates differed substantially with regards to the host immune response. Especially isolate 33 resulted in severe infiltration of inflammatory cells in the basal region of the valve along with pronounced fibrinous exudates and bacterial vegetations (Figure 4a). Only in this isolate the gene for the superantigen Sec, which is known to induce fulminant cytokine release and T-cell activation, was present. Other studies have also discussed the importance of superantigens for the development of endocarditis [30,42]. In addition, strain 33 showed *in vitro* extremely low levels of factors such as Efb, Cna and SpA that are capable to suppress complement activation. Thus, the complement system is probably fully activated by this isolate, which could explain the observed mobilization of large amounts of neutrophils. On the one hand, this protects the host from invading bacteria, but on the other hand, it may result in overwhelming inflammation and host tissue damage [43]. Similarly, in samples from patients with IE, tissue destruction could be associated with neutrophils and neutrophil proteases [44,45]. All these mechanisms could explain why isolate 33, which had non-aggressive characteristics *in vitro* (low invasion, low cytotoxicity, low gene expression of *agr* and *sarA*), led to the strongest and fastest tissue destruction and infiltration of immune cells *in vivo*, in comparison to strain 17 and 30.

In contrast, lower immune cell infiltration was observed in infection with strain 17. A possible explanation might be the high gene expression of *aur* and *efb* in this strain, which might impair the complement system [12]. Another potential factor might be the destructive effect of *S. aureus* toxins such as Hla and PSMα on immune cells. In addition, the *S. aureus* adhesins ClfA, FnBPA and Eap, which were highly expressed in strain 17, enable binding to the extracellular matrix as well as binding to and activation of platelets. The importance of these factors for the development of endocarditis has also been shown previously both in animal experiments and in clinical studies [15,31,46–49]. Differences between the three *S. aureus* strains were additionally found in the expression profiles of *sak, scn* and *chp*. However, a potential role of these factors in our experimental mouse setting is unlikely as these factors are human specific [50]. For the induction of bacterial vegetations, it is well known that the exposure of extracellular matrix to the bloodstream leads to the deposition of fibrin and platelets [3,51]. This cascade results in fibrinous clots being able to envelope large amounts of bacteria. These histopathological findings correlated well with the predicted activity of regenerative pathways such as angiogenesis, vasculogenesis and the organization of the extracellular matrix (Figure 5c). A corresponding enrichment analysis, taking into account the top three enriched pathways per isolate, confirmed this prediction (Figure 5d). Inflammation and angiogenesis are strongly connected to each other and mainly trigger and control tissue regeneration. Together with pattern recognition molecules such as cytokines, chemokines and growth factor, leukocytes regulate neovascularization at the site of vascular damage and tissue injury [52]. Investigations of gene expression profiles of aortic valves infected with isolate 33 showed in particular high expression levels in *CCL2* (*MCP-1), CCL5* (*RANTES), IL1a, IL6* (supplementary table 4 and 5), which to some extent reflects the *in vitro* results (Figure 2b). Recently, it was revealed that immunomodulatory and proangiogenic factors such as MCP-1 [52] and RANTES [53] improve the regeneration of the injured tissue. In addition, our observation of functional destruction of the valve after infection with isolate 33 confirmed earlier reports, describing that genes regulating immune cell recruitment, angiogenesis and tissue remodeling are expressed in IE [54]. In contrast, valvular tissue infected with isolates 17 and 30 showed substantially less expression of these immunomodulatory recognition molecules, as compared to isolate 33. In addition, less immune cell invasion was observed, and no activation or enrichment of pathways connected to tissue regeneration could be identified for these two isolates. Instead, several pathways connected to movement, migration, recruitment and adhesion of immune cells and endothelial cells were highly upregulated, indicating that the inflammatory stage and progression might be delayed in comparison to isolate 33. The pathogenicity of isolates which have been classified as non-aggressive *in vitro*, has also been previously demonstrated in sepsis/osteomyelitis models [55].

## Conclusion

The results obtained *in vitro* cannot be translated directly to *in vivo* conditions. Although the selected isolates differed distinctively in the performed *in vitro* assays, all isolates resulted in a similar clinical picture *in vivo*, particularly with similar bacterial loads in all tissues examined. A longitudinal study design covering a longer observation period might result in differential findings, especially since the analysis of activated pathways of immune response showed clear differences in the manifestation of the infection due to the distinct virulence patterns of the three isolates. In patients, the different ways of host response may also be altered depending on the immune status. Therefore, our data advise to pursue comprehensive evaluation approaches of the pathogenic capabilities of bacteria, including the assessment of disease-relevant pathogenic interactions and pathways of the immune response.

## Supplementary Material

Supplemental Material

## Data Availability

All datasets generated for this study in the article can be accessed here: 10.6084/m9.figshare.14974257.
