# Supplementary material for: Host-pathogen interactions of clinical S. aureus isolates to induce infective endocarditis
Source: Virulence. 2021 Sep 7;12(1):2073–87. doi: 10.1080/21505594.2021.1960107 (PMC8425731; doi:10.1080/21505594.2021.1960107)
Supplement: Supplemental Material [file KVIR_A_1960107_SM2445.zip › supplementary/11-Schwarz-SUPPLEMENTARY-Table-2-5-20210702clean.docx]

**SUPPLEMENTARY MATERIAL: Tables 2 - 5**

Supplementary table 2: spa typing and susceptibility to antibiotics

| **isolate number** | **spa-type** | **MSSA/MRSA** | **material** |
| --- | --- | --- | --- |
| 2 | t008 | MSSA | blood culture |
| 5 | t0091 | MSSA | blood culture |
| 11 | t095 | MSSA | blood culture |
| 12 | t156 | MSSA | blood culture |
| 15 | t091 | MSSA | blood culture |
| 16 | t003 | MRSA | blood culture |
| 17 | t159 | MSSA | blood culture |
| 18 | t223 | MSSA | blood culture |
| 20 | t3275 | MSSA | blood culture |
| 21 | t514 | MSSA | blood culture |
| 22 | t024 | MSSA | blood culture |
| 23 | t2576 | MRSA | blood culture |
| 24 | t032 | MRSA | blood culture |
| 25 | t046 | MSSA | blood culture |
| 27 | t091 | MSSA | blood culture |
| 28 | t1460 | MSSA | blood culture |
| 30 | t092 | MSSA | blood culture |
| 32 | t190 | MSSA | blood culture |
| 33 | t032 | MRSA | tissue |
| 34 | t005 | MSSA | blood culture |
| 35 | t7126 | MSSA | blood culture |
| 36 | t18220 | MSSA | blood culture |

Supplementary table 3: Primer Sequences

| **Gene name** | **Primer** | | **Reference** |
| --- | --- | --- | --- |
| *agrA* | Forward  Reverse | 5′-AACTGCACATACACGCTTACA-3′  5′-GGCAATGAGTCTGTGAGATTT-3′ | ^1^ |
| *aur* | Forward  Reverse | 5′-ACCGTGTGTTAATTCGTGTGCTA-3′  5′-ATGGTCGCACATTCACAAGTTT-3′ | ^1^ |
| *CCL5* | Forward  Reverse | 5′-CAGTGGCAAGTGCTCCAACC-3′  5′-CCATCCTAGCTCATCTCCAAAGAGT-3′ | ^1^ |
| *chp* | Forward  Reverse | 5′-AATAGTGGTCTTCCTACAACA-3′  5′-CAGCAAGTGGTGTATTCAG-3′ | ^2^ |
| *clfA* | Forward  Reverse | 5′-GAATCAGCTCCACAGAGTACAG-3′  5′-TCTCATTCTAGGCGCACTTG-3′ | ^1^ |
| *cna* | Forward  Reverse | 5′-AAAGCGTTGCCTAGTGGAGA-3′  5′-AGTGCCTTCCCAAACCTTTT-3′ | ^1^ |
| *CXCL10* | Forward  Reverse | 5′-GGAACCTCCAGTCTCAGCACCA-3′  5′-AGACATCTCTTCTCACCCTTC-3′ | ^1^ |
| *eap* | Forward  Reverse | 5′-AGTCATTGATTACAACAA-3′  5′-CTTATTAAATGTTAAGCTTG-3‘ | ^1^ |
| *efb* | Forward  Reverse | 5′-AACCAGCAGCGAAAACTGAT-3′  5′-CTGCCTTTTGTGCTTTTCTG-3′ | ^3^ |
| *fnbA* | Forward  Reverse | 5′-ACAAGTTGAAGTGGCACAGCC-3′  5′-CCGCTACATCTGCTGATCTTGTC-3′ | ^1^ |
| *GAPDH *** | Forward  Reverse | 5′-GCAAATTTCCATGGCACCGT-3′  5′-GCCCCACTTGATTTTGGAGG-3′ | ^1^ |
| *gyrB ** | Forward  Reverse | 5′-aattgaagcaggctatgtgt-3′  5′-atagaccattttggtgttgg-3′ | ^1^ |
| *hla* | Forward  Reverse | 5′-caactgataaaaaagtaggctggaaagtgat-3′  5′-ctggtgaaaaccctgaagataatagag-3′ | ^1^ |
| *ICAM-1* | Forward  Reverse | 5′-ACCTCCCCACCCACATACATTT-3′  5′-GGCATAGCTTGGGCATATTCC-3′ | ^1^ |
| *IL-6* | Forward  Reverse | 5′-AGAGGCACTGGCAGAAAACAAC-3′  5′-AGGCAAGCTTCCTCATTGAATCC-3′ | ^1^ |
| *psmα* | Forward  Reverse | 5′-GCCATTCACATGGAATTCGT-3′  5′- CAATAGCCATCGTTTTGTCCT-3′ | ^4^ |
| *saeR* | Forward  Reverse | 5'-ATTTACGCCTTAACTTTAGGTG-3'  5'-AACTGGTTGATGATGGTATTT-3' | ^1^ |
| *sak* | Forward  Reverse | 5′-TGTCGAATGGGCATTAGATGCGAC-3′  5′-GTGACTTCGATCTTTGCGCTTG-3′ | ^5^ |
| *sarA* | Forward  Reverse | 5'-ACATGGCAATTACAAAAATCAATGAT-3'  5'-TCTTTCTCTTTGTTTTCGCTGATG-3' | ^1^ |
| *scn* | Forward  Reverse | 5′-AGCACAAGCTTGCCAACATCG-3′  5′-TTAATATTTACTTTTTAGTGC-3′ | ^6^ |
| *sea* | Forward  Reverse | 5′-GGTTATCAATGTGCGGGTGG-3′  5′-CGGCACTTTTTTCTCTTCGG-3′ | ^7^ |
| *seb* | Forward  Reverse | 5′-GTATGGTGGTGTAACTGAGC-3′  5′-CCAAATAGTGACGAGTTAGG-3′ | ^7^ |
| *sec* | Forward  Reverse | 5′-AGATGAAGTAGTTGATGTGTATGG-3′  5′-CACACTTTTAGAATCAACCG-3′ | ^7^ |
| *sigB* | Forward  Reverse | 5′-atgtacgtttattgaaggattg-3′  5′-taatttcttaattgccgttctc-3′ | ^1^ |
| *spa* | Forward  Reverse | 5′-cagataacaaattagctgataaaaacat-3′  5′-ctaaggctaatgataatccaccaaatac-3′ | ^1^ |
| *tst* | Forward  Reverse | 5′-ACCCCTGTTCCCTTATCATC-3′  5′-TTTTCAGTATTTGTAACGCC-3′ | ^7^ |
| *vWbp* | Forward  Reverse | 5′-CGACAAGAAACTTGTGGTTTCTGCACC-3′  5′-TGACGCTCAACTGTAGGCATTGGT-3′ | ^5^ |
| *β-actin *** | Forward  Reverse | 5′-AACTGGAACGGTGAAGGTG-3′  5′-CTGTGTGGACTTGGGAGAGG-3′ | ^1^ |

* housekeeping gene *S. aureus,* ** housekeeping genes host cell (EA.hy926)

References of supplementary table 3: Primer Sequences

1. Kalinka J, Hachmeister M, Geraci J, Sordelli D, Hansen U, Niemann S, Oetermann S, Peters G, Löffler B, Tuchscherr L. Staphylococcus aureus isolates from chronic osteomyelitis are characterized by high host cell invasion and intracellular adaptation, but still induce inflammation. *Int J Med Microbiol* 2014;**304**:1038–1049.

2. Delauné A, Dubrac S, Blanchet C, Poupel O, Mäder U, Hiron A, Leduc A, Fitting C, Nicolas P, Cavaillon J-M, Adib-Conquy M, Msadek T. The WalKR system controls major staphylococcal virulence genes and is involved in triggering the host inflammatory response. *Infect Immun* 2012;**80**:3438–3453.

3. Kitichalermkiat A, Katsuki M, Sato J, Sonoda T, Masuda Y, Honjoh K-I, Miyamoto T. Effect of epigallocatechin gallate on gene expression of Staphylococcus aureus. *J Glob Antimicrob Resist* 2020;**22**:854–859.

4. Jordan PM, Gerstmeier J, Pace S, Bilancia R, Rao Z, Börner F, Miek L, Gutiérrez-Gutiérrez Ó, Arakandy V, Rossi A, Ialenti A, González-Estévez C, Löffler B, Tuchscherr L, Serhan CN, Werz O. Staphylococcus aureus-Derived α-Hemolysin Evokes Generation of Specialized Pro-resolving Mediators Promoting Inflammation Resolution. *Cell Rep* 2020;**33**:108247.

5. Walker JN, Crosby HA, Spaulding AR, Salgado-Pabón W, Malone CL, Rosenthal CB, Schlievert PM, Boyd JM, Horswill AR. The Staphylococcus aureus ArlRS two-component system is a novel regulator of agglutination and pathogenesis. *PLoS Pathog* 2013;**9**:e1003819.

6. Ahmadrajabi R, Layegh-Khavidaki S, Kalantar-Neyestanaki D, Fasihi Y. Molecular analysis of immune evasion cluster (IEC) genes and intercellular adhesion gene cluster (ICA) among methicillin-resistant and methicillin-sensitive isolates of Staphylococcus aureus. *J Prev Med Hyg* 2017;**58**:E308-E314.

7. Elsherif HM, Helal ZH, El-Ansary MR, Fahmy ZA, Eltayeb WN, Radwan S, Aboshanab KM. Staphylococcal Enterotoxins and Toxic Shock Syndrome Toxin-1 and Their Association among Bacteremic and Infective Endocarditis Patients in Egypt. *Biomed Res Int* 2020;**2020**:6981095.

Supplementary table 4: Gene Array Mouse Extracellular Matrix & Adhesion Molecules.

| 2(-ΔΔCt) fold change | |  | | | ***S. aureus* strains** | |
| --- | --- | --- | --- | --- | --- | --- |
| **Gene name** | **SHAM** | | **17** | **30** | | **33** |
| Adamts 1 | 2.10 | | 3.35 | 0.12 | | 10.07 |
| Adamts 2 | 0.38 | | 1.55 | 1.01 | | 4.85 |
| Adamts 5 | 0.23 | | 1.61 | 0.79 | | 3.46 |
| Adamts 8 | 1.26 | | 4.56 | 3.08 | | 39.12 |
| Cd44 | 0.77 | | 4.20 | 5.85 | | 21.58 |
| Cdh1 | 21.12 | | 2.07 | 7.28 | | 193.66 |
| Cdh2 | 0.18 | | 1.88 | 0.73 | | 3.81 |
| Cdh3 | 0.12 | | 2.07 | 1.20 | | 4.22 |
| Cdh4 | 0.10 | | 0.83 | 0.29 | | 2.24 |
| Cntn1 | 18.00 | | 7.46 | 1.01 | | 4.22 |
| Col1a1 | 0.48 | | 1.90 | 1.51 | | 4.94 |
| Col2a1 | 19.35 | | 2.07 | 4.14 | | 12.37 |
| Col3a1 | 0.93 | | 2.08 | 1.89 | | 4.73 |
| Col4a1 | 0.66 | | 4.30 | 2.39 | | 12.40 |
| Col4a2 | 0.42 | | 3.45 | 1.81 | | 8.65 |
| Col4a3 | 0.24 | | 1.13 | 0.57 | | 3.88 |
| Col5a1 | 0.39 | | 1.35 | 1.21 | | 4.01 |
| Col6a1 | 0.25 | | 1.57 | 0.99 | | 4.48 |
| Ctgf | 1.54 | | 4.93 | 3.35 | | 9.67 |
| Ctnna1 | 0.19 | | 1.66 | 0.78 | | 4.43 |
| Ctnna2 | 83.91 | | 9.17 | 12.63 | | 65.89 |
| Ctnnb1 | 0.17 | | 1.23 | 0.65 | | 4.94 |
| Ecm1 | 0.57 | | 2.69 | 1.93 | | 18.05 |
| Emilin1 | 0.00 | | 0.00 | 0.00 | | 0.02 |
| Entpd1 | 0.01 | | 2.50 | 1.48 | | 9.52 |
| Fbln1 | 0.10 | | 1.18 | 0.60 | | 4.39 |
| Fn1 | 1.20 | | 2.87 | 2.64 | | 10.28 |
| Hapln1 | 0.66 | | 0.55 | 0.21 | | 21.98 |
| Hc | 24.90 | | 9.82 | 31.26 | | 155.93 |
| Icam1 | 0.38 | | 7.12 | 5.79 | | 74.22 |
| Itga2 | 0.64 | | 2.61 | 3.13 | | 19.71 |
| Itga3 | 0.27 | | 2.02 | 0.96 | | 4.11 |
| Itga4 | 0.45 | | 2.12 | 2.43 | | 12.01 |
| Itga5 | 0.76 | | 3.86 | 2.98 | | 12.95 |
| Itgae | 1.11 | | 1.56 | 2.63 | | 11.68 |
| Itgal | 0.72 | | 6.88 | 7.44 | | 37.09 |
| Itgam | 3.67 | | 8.19 | 11.04 | | 54.13 |
| Itgav | 0.00 | | 1.55 | 1.07 | | 4.46 |
| Itgax | 0.00 | | 3.09 | 0.46 | | 9.02 |
| Itgb1 | 0.21 | | 1.27 | 0.79 | | 4.51 |
| Itgb2 | 1.62 | | 5.78 | 6.46 | | 41.74 |
| Itgb3 | 0.69 | | 4.65 | 2.91 | | 19.15 |
| Itgb4 | 0.24 | | 1.42 | 1.60 | | 4.11 |
| Lama1 | 2.03 | | 3.37 | 1.01 | | 4.22 |
| Lama2 | 0.17 | | 1.39 | 0.41 | | 2.68 |
| Lama3 | 0.29 | | 1.36 | 0.68 | | 4.10 |
| Lamb2 | 0.17 | | 1.64 | 0.63 | | 3.58 |
| Lamb3 | 0.11 | | 1.41 | 0.34 | | 0.00 |
| Lamc1 | 0.23 | | 1.92 | 0.92 | | 4.83 |
| mmp10 | 29.01 | | 2.07 | 26.72 | | 8.87 |
| mmp11 | 0.12 | | 1.17 | 0.47 | | 2.26 |
| mmp12 | 3.71 | | 4.64 | 8.09 | | 0.28 |
| mmp13 | 0.48 | | 0.43 | 1.40 | | 9.26 |
| mmp14 | 1.10 | | 4.54 | 4.41 | | 15.01 |
| mmp15 | 0.00 | | 0.87 | 0.42 | | 2.22 |
| mmp1a | 0.17 | | 2.38 | 0.84 | | 3.89 |
| mmp2 | 0.15 | | 1.48 | 0.68 | | 3.79 |
| mmp3 | 0.79 | | 5.31 | 3.91 | | 20.00 |
| mmp7 | 13.74 | | 2.07 | 8.15 | | 4.22 |
| mmp8 | 16.85 | | 147.02 | 107.64 | | 1582.17 |
| mmp9 | 4.88 | | 7.61 | 3.77 | | 36.52 |
| Ncam1 | 0.43 | | 3.20 | 1.81 | | 7.18 |
| Ncam2 | 1.43 | | 1.80 | 0.24 | | 23.80 |
| PECAM1 | 0.23 | | 2.16 | 1.06 | | 6.70 |
| Postn | 0.30 | | 0.69 | 1.00 | | 2.83 |
| Sele | 1.20 | | 21.92 | 30.36 | | 327.44 |
| Sell | 2.85 | | 11.45 | 9.55 | | 170.18 |
| Selp | 12.39 | | 70.10 | 117.94 | | 203.61 |
| Sgce | 0.29 | | 2.03 | 0.96 | | 8.05 |
| Sparc | 0.38 | | 2.00 | 1.09 | | 4.44 |
| Spock1 | 1.09 | | 0.12 | 0.06 | | 0.61 |
| Spp1 | 119.07 | | 17.15 | 478.04 | | 207.04 |
| Syt1 | 1.98 | | 2.07 | 1.01 | | 4.22 |
| Tgfbi | 0.86 | | 2.34 | 3.32 | | 10.57 |
| Thbs1 | 0.26 | | 8.02 | 14.47 | | 78.31 |
| Thbs2 | 0.28 | | 2.34 | 0.84 | | 4.79 |
| Thbs3 | 0.19 | | 1.90 | 0.98 | | 8.05 |
| timp1 | 40.87 | | 42.67 | 121.55 | | 242.51 |
| timp2 | 0.18 | | 1.59 | 0.88 | | 5.02 |
| timp3 | 0.28 | | 3.60 | 1.19 | | 8.85 |
| tnc | 25.78 | | 22.25 | 126.83 | | 74.29 |
| Vcam1 | 0.20 | | 1.98 | 2.05 | | 23.95 |
| Vcan | 0.72 | | 8.06 | 5.78 | | 34.36 |
| Vtn | 0.22 | | 1.31 | 0.65 | | 3.02 |

Supplementary table 5: Gene Array Inflammatory Response and Autoimmunity.

| 2(-ΔΔCt) fold change | |  | | ***S. aureus* strains** | | |
| --- | --- | --- | --- | --- | --- | --- |
| **Gene name** | **SHAM** | | **17** | | **30** | **33** |
| Bcl6 | 0.43 | | 1.48 | | 1.60 | 1.67 |
| C3 | 0.32 | | 2.75 | | 1.53 | 4.01 |
| C3ar1 | 0.15 | | 0.43 | | 0.40 | 0.50 |
| C4b | 0.12 | | 2.44 | | 0.54 | 1.09 |
| Ccl1 | 3.12 | | 1.51 | | 1.52 | 2.35 |
| Ccl11 | 0.04 | | 0.15 | | 0.15 | 0.46 |
| Ccl12 | 0.04 | | 0.16 | | 0.42 | 1.33 |
| Ccl17 | 0.24 | | 0.93 | | 0.81 | 3.84 |
| ccl19 | 0.07 | | 1.34 | | 0.67 | 2.68 |
| Ccl2 | 0.58 | | 3.52 | | 3.97 | 17.58 |
| Ccl22 | 422.68 | | 139.68 | | 322.09 | 1019.32 |
| Ccl24 | 4.91 | | 5637.76 | | 3.54 | 7.58 |
| Ccl25 | 0.39 | | 0.74 | | 0.70 | 0.89 |
| Ccl3 | 0.54 | | 1.90 | | 1.89 | 9.97 |
| Ccl4 | 0.78 | | 6.11 | | 6.07 | 32.19 |
| Ccl5 | 0.12 | | 0.76 | | 0.40 | 2.24 |
| Ccl7 | 0.08 | | 0.41 | | 0.71 | 2.88 |
| Ccl8 | 0.11 | | 0.75 | | 0.58 | 0.94 |
| Ccr1 | 0.32 | | 0.93 | | 0.57 | 1.47 |
| Ccr2 | 1922.03 | | 16.50 | | 49.18 | 42.15 |
| Ccr3 | 1047.16 | | 29.79 | | 34.65 | 49.48 |
| Ccr4 | 1.38 | | 7.22 | | 0.00 | 0.03 |
| Ccr7 | 0.48 | | 1.32 | | 0.86 | 1.27 |
| Cd14 | 0.51 | | 4.13 | | 1.52 | 7.25 |
| Cd40 | 0.31 | | 2.43 | | 0.03 | 2.52 |
| Cd40lg | 11.53 | | 4.67 | | 37.22 | 7.61 |
| Cebpd | 0.15 | | 1.92 | | 0.53 | 1.46 |
| Crp | 0.01 | | 0.50 | | 0.00 | 0.41 |
| Cdf1 | 0.10 | | 1.10 | | 0.44 | 1.18 |
| Cxcl1 | 0.08 | | 2.16 | | 2.22 | 11.31 |
| Cxcl10 | 0.66 | | 12.97 | | 16.23 | 72.08 |
| Cxcl11 | 19.34 | | 0.13 | | 6.04 | 8.73 |
| Cxcl2 | 3564.59 | | 688.65 | | 460.81 | 2177.56 |
| Cxcl3 | 6061.08 | | 20385.92 | | 7385.58 | 51370.32 |
| Cxcl5 | 1.04 | | 12.38 | | 28.64 | 61.81 |
| Cxcl9 | 0.03 | | 2.16 | | 1.89 | 6.34 |
| Cxcr1 | 0.00 | | 0.00 | | 0.00 | 0.00 |
| Cxcr2 | 21.65 | | 42.56 | | 25.99 | 127.90 |
| Cxcr4 | 0.01 | | 0.06 | | 0.02 | 0.03 |
| Fasl | 0.03 | | 0.03 | | 0.02 | 0.41 |
| Fos | 0.42 | | 1.35 | | 0.54 | 4.19 |
| Ifng | 9.17 | | 127.22 | | 39.73 | 413.32 |
| IL10 | 220.18 | | 1174.14 | | 609.82 | 1385.13 |
| IL10rb | 0.07 | | 0.43 | | 0.01 | 0.31 |
| IL17a | 1.30 | | 0.94 | | 0.94 | 1.53 |
| IL18 | 492.30 | | 55.04 | | 43.78 | 66.75 |
| IL1a | 2.06 | | 19.00 | | 20.46 | 138.93 |
| IL1b | 1.33 | | 18.96 | | 11.07 | 63.11 |
| IL1r | 0.25 | | 1.82 | | 1.07 | 1.69 |
| IL1rap | 0.11 | | 0.55 | | 0.24 | 0.64 |
| IL1rn | 1574.86 | | 4655.73 | | 3416.19 | 12805.14 |
| IL22 | 10.22 | | 1.51 | | 6.15 | 21.04 |
| IL23a | 34.24 | | 7.39 | | 5.93 | 13.95 |
| IL23r | 10.80 | | 3.69 | | 4.39 | 16.16 |
| IL5 | 262.72 | | 109.86 | | 261.69 | 1317.58 |
| IL6 | 131.44 | | 4603.06 | | 4148.95 | 19833.37 |
| IL6ra | 1077.88 | | 0.04 | | 50.31 | 115.78 |
| IL7 | 0.02 | | 0.00 | | 0.05 | 0.08 |
| IL9 | 9.19 | | 1.36 | | 6.26 | 2.21 |
| Itgb2 | 4821.66 | | 26416.85 | | 11979.58 | 22930.29 |
| Kng1 | 12.71 | | 1.51 | | 1.52 | 2.46 |
| Lta | 11.11 | | 1.53 | | 4.84 | 51.61 |
| Ltb | 0.13 | | 0.79 | | 0.31 | 1.80 |
| Ly96 | 0.05 | | 0.30 | | 0.15 | 0.27 |
| Myd88 | 0.10 | | 0.79 | | 0.41 | 1.12 |
| Nfkb1 | 4822.44 | | 359.98 | | 88.45 | 192.18 |
| Nos2 | 0.03 | | 0.15 | | 0.06 | 0.10 |
| Nr3c1 | 0.02 | | 0.13 | | 0.04 | 0.09 |
| Ptgs2 | 0.19 | | 1.34 | | 0.65 | 2.93 |
| Ripk2 | 0.19 | | 1.19 | | 0.82 | 2.06 |
| Sele | 0.55 | | 8.52 | | 7.16 | 23.29 |
| Tirap | 0.10 | | 1.47 | | 0.19 | 0.69 |
| Tlr1 | 1.80 | | 4.81 | | 5.00 | 16.89 |
| Tlr2 | 0.09 | | 0.86 | | 0.62 | 2.04 |
| Tlr3 | 0.02 | | 0.16 | | 0.11 | 0.41 |
| Tlr4 | 0.31 | | 1.55 | | 0.61 | 1.23 |
| Tlr5 | 0.14 | | 0.64 | | 0.24 | 0.34 |
| Tlr7 | 0.15 | | 0.48 | | 0.33 | 0.45 |
| Tlr9 | 0.06 | | 0.14 | | 0.33 | 0.45 |
| Tnf | 113.44 | | 51.91 | | 43.24 | 227.47 |
| Tnfsf14 | 244.27 | | 310.82 | | 79.12 | 585.40 |
| Tollip | 0.09 | | 0.60 | | 0.17 | 0.48 |
