# Supplementary material for: Host-pathogen interactions of clinical S. aureus isolates to induce infective endocarditis
Source: Virulence. 2021 Sep 7;12(1):2073–87. doi: 10.1080/21505594.2021.1960107 (PMC8425731; doi:10.1080/21505594.2021.1960107)
Supplement: Supplemental Material [file KVIR_A_1960107_SM2445.zip › supplementary/14-Schwarz-SUPPLENTARY-Table-1-2021-07-02-Revision-2clean.docx]

| Virulence factor | Function | Reference |
| --- | --- | --- |
| **Regulator systems** | | |
| accessory gene regulator (Agr) | - quorum-sensing system - activation leads to expression of exotoxins | ^1,2^ |
| staphylococcal accessory regulator (SarA) | - upregulation of agr - resulting in enhanced expression of toxins - expression of protein A (spa) | ^1,2^ |
| S. aureus exoprotein expression (SAE) | - encodes the SaeRS two component system - drives the expression of important exoproteins (e.g., coagulase, a-toxin, TSST-1, different adhesins) | ^2^ |
| Alternative Sigma Factor (SigB) | - stress and stationary-phase-specific global regulator which is directly and indirectly involved in the expression of virulence genes - inhibits agr system | ^2,3^ |
| **MSCRAMMs (Microbial Surface Components Recognizing Adhesive Matrix Molecules)** | | |
| Clumping factor A (ClfA) | - adhesion to fibrinogen - immune evasion by binding soluble fibrinogen - mediates binding to and activation of platelets - degradation of complement C3b | ^4–9^ |
| Fibronectin-binding proteins A (FnBPA) | - binding to extracellular matrix - mediates host cell invasion - mediates binding to and activation of platelets | ^9–12^ |
| Collagen adhesin (Cna) | - Binding to collagen - Inhibition of the classical complement pathway | ^13,14^ |
| Staphylococcal protein A (SpA) | - Binding to vWF - binding of IgG Fc fragments and thereby inhibiting complement fixation by the classical pathway - binding of Fab regions of B-cells and thereby inducing B-cell death and preventing the production of antibody specific for *S. aureus* | ^15–17^ |
| **SERAMs (Secretable Expanded Repertoire Adhesive Molecules)** | | |
| Extracellular adherence protein (Eap)/ MHC analogous protein (Map) | - Mediates adhesion to matrix proteins - promotes uptake of *S. aureus* by eukaryotic cells - inhibits leukocyte recruitment - decreases phagocytic activity - activates platelets - binding of host DNA and thereby supressing the formation of neutrophil extracellular traps | ^18–21^ |
| von Willebrand factor binding protein (vWbp) | - Binding of von Willebrand factor (vWF) - vWbp activates a direct interaction between ClfA and vWF - activator of prothrombin which converts fibrinogen to fibrin, leading to blood clotting | ^22–27^ |
| Coagulase (Coa) | - activator of prothrombin which converts fibrinogen to fibrin, leading to blood clotting | ^25–28^ |
| Extracellular fibrinogen binding protein (Efb) | - Binding of fibrinogen, binding to platelets and inhibition of platelet aggregation - binds to C3b and inhibits both complement-mediated lysis and opsonophagocytosis | ^29–32^ |
| **Immune evasion** | | |
| Staphylokinase (SAK) | - binding to plasminogen results in the formation of active plasmin, which digests fibrin clots - binding to defensins and neutralizing their bactericidal ability - SAK-induced plasmin activity can degrade opsonizing complement components IgG and C3b | ^33,34^ |
| chemotaxis inhibitory protein of *S. aureus* (CHIPS)  chemotaxis inhibitory protein of *S. aureus* (CHIPS) | - Impairs the response of neutrophils and monocytes to formylated peptides and C5a - inhibits neutrophil recruitment - cellular activation by C5a is blocked | ^35,36^  ^35,36^ |
| Staphylococcal complement inhibitor (SCIN) | - blocks all complement pathways: the lectin, classical and alternative pathway - prevents phagocytosis and C5a production | ^36,37^ |
| **Toxins and proteases** | | |
| a-toxin (Hla) | - effects on e.g., platelets, monocytes, neutrophils, T cells, endothelial cells, dependent on the relative concentration of hla: from activation to lysis | ^38,39^ |
| Phenol soluble modulins (PSMs) | - stimulate the release of pro-inflammatory cytokines - induce migration and activation in macrophages and neutrophils - lyse human neutrophils - facilitate phagosomal escape | ^40–43^ |
| Enterotoxins (Sea, Seb, Sec and TSST-1) | - Superantigens - binding to various MHC II types and non-specific activation of T-cells resulting in massive cytokine release | ^44,45^ |
| Aureolysin (Aur) | - Activates serine proteases by cleavage of proenzyme form - complement inhibitor by cleavage of the central complement protein C3 | ^46,47^ |

References of supplementary table 1

1. Cheung AL, Bayer AS, Zhang G, Gresham H, Xiong Y-Q. Regulation of virulence determinants in vitro and in vivo in Staphylococcus aureus. *FEMS Immunol Med Microbiol* 2004;**40**:1–9.

2. Jenul C, Horswill AR. Regulation of Staphylococcus aureus Virulence. In: Fischetti VA, Novick RP, Ferretti JJ, Portnoy DA, Braunstein M, Rood JI. *Gram-positive pathogens*. 3rd edition. Washington, DC: ASM Press, 2019:669–686.

3. Kullik I, Giachino P, Fuchs T. Deletion of the alternative sigma factor sigmaB in Staphylococcus aureus reveals its function as a global regulator of virulence genes. *J Bacteriol* 1998;**180**:4814–4820.

4. Foster TJ, Geoghegan JA, Ganesh VK, Hook M. Adhesion, invasion and evasion: the many functions of the surface proteins of Staphylococcus aureus. *Nature reviews. Microbiology* 2014;**12**:49–62.

5. Herman-Bausier P, Labate C, Towell AM, Derclaye S, Geoghegan JA, Dufrêne YF. Staphylococcus aureus clumping factor A is a force-sensitive molecular switch that activates bacterial adhesion. *PNAS* 2018;**115**:5564–5569.

6. Siboo IR, Cheung AL, Bayer AS, Sullam PM. Clumping factor A mediates binding of Staphylococcus aureus to human platelets. *Infect Immun* 2001;**69**:3120–3127.

7. Niemann S, Spehr N, van Aken H, Morgenstern E, Peters G, Herrmann M, Kehrel BE. Soluble fibrin is the main mediator of Staphylococcus aureus adhesion to platelets. *Circulation* 2004;**110**:193–200.

8. Loughman A, Fitzgerald JR, Brennan MP, Higgins J, Downer R, Cox D, Foster TJ. Roles for fibrinogen, immunoglobulin and complement in platelet activation promoted by Staphylococcus aureus clumping factor A. *Mol Microbiol* 2005;**57**:804–818.

9. Que Y-A, Haefliger J-A, Piroth L, François P, Widmer E, Entenza JM, Sinha B, Herrmann M, Francioli P, Vaudaux P, Moreillon P. Fibrinogen and fibronectin binding cooperate for valve infection and invasion in Staphylococcus aureus experimental endocarditis. *J Exp Med* 2005;**201**:1627–1635.

10. Speziale P, Pietrocola G. The Multivalent Role of Fibronectin-Binding Proteins A and B (FnBPA and FnBPB) of Staphylococcus aureus in Host Infections. *Front Microbiol* 2020;**11**:2054.

11. Sinha B, François PP, Nüsse O, Foti M, Hartford OM, Vaudaux P, Foster TJ, Lew DP, Herrmann M, Krause KH. Fibronectin-binding protein acts as Staphylococcus aureus invasin via fibronectin bridging to integrin alpha5beta1. *Cell Microbiol* 1999;**1**:101–117.

12. Fitzgerald JR, Loughman A, Keane F, Brennan M, Knobel M, Higgins J, Visai L, Speziale P, Cox D, Foster TJ. Fibronectin-binding proteins of Staphylococcus aureus mediate activation of human platelets via fibrinogen and fibronectin bridges to integrin GPIIb/IIIa and IgG binding to the FcgammaRIIa receptor. *Mol Microbiol* 2006;**59**:212–230.

13. Zong Y, Xu Y, Liang X, Keene DR, Höök A, Gurusiddappa S, Höök M, Narayana SVL. A 'Collagen Hug' model for Staphylococcus aureus CNA binding to collagen. *EMBO J* 2005;**24**:4224–4236.

14. Kang M, Ko Y-P, Liang X, Ross CL, Liu Q, Murray BE, Höök M. Collagen-binding microbial surface components recognizing adhesive matrix molecule (MSCRAMM) of Gram-positive bacteria inhibit complement activation via the classical pathway. *J Biol Chem* 2013;**288**:20520–20531.

15. Hartleib J, Köhler N, Dickinson RB, Chhatwal GS, Sixma JJ, Hartford OM, Foster TJ, Peters G, Kehrel BE, Herrmann M. Protein A is the von Willebrand factor binding protein onStaphylococcus aureus. *Blood* 2000;**96**:2149–2156.

16. Atkins KL, Burman JD, Chamberlain ES, Cooper JE, Poutrel B, Bagby S, Jenkins ATA, Feil EJ, van den Elsen JMH. S. aureus IgG-binding proteins SpA and Sbi: host specificity and mechanisms of immune complex formation. *Mol Immunol* 2008;**45**:1600–1611.

17. Goodyear CS, Silverman GJ. Death by a B cell superantigen: In vivo VH-targeted apoptotic supraclonal B cell deletion by a Staphylococcal Toxin. *J Exp Med* 2003;**197**:1125–1139.

18. Chavakis T, Hussain M, Kanse SM, Peters G, Bretzel RG, Flock JI, Herrmann M, Preissner KT. Staphylococcus aureus extracellular adherence protein serves as anti-inflammatory factor by inhibiting the recruitment of host leukocytes. *Nat Med* 2002;**8**:687–693.

19. Harraghy N, Hussain M, Haggar A, Chavakis T, Sinha B, Herrmann M, Flock J-I. The adhesive and immunomodulating properties of the multifunctional Staphylococcus aureus protein Eap. *Microbiology (Reading, Engl )* 2003;**149**:2701–2707.

20. Bertling A, Niemann S, Hussain M, Holbrook L, Stanley RG, Brodde MF, Pohl S, Schifferdecker T, Roth J, Jurk K, Muller A, Lahav J, Peters G, Heilmann C, Gibbins JM, Kehrel BE. Staphylococcal extracellular adherence protein induces platelet activation by stimulation of thiol isomerases. *Arterioscler Thromb Vasc Biol* 2012;**32**:1979–1990.

21. Eisenbeis J, Saffarzadeh M, Peisker H, Jung P, Thewes N, Preissner KT, Herrmann M, Molle V, Geisbrecht BV, Jacobs K, Bischoff M. The Staphylococcus aureus Extracellular Adherence Protein Eap Is a DNA Binding Protein Capable of Blocking Neutrophil Extracellular Trap Formation. *Front Cell Infect Microbiol* 2018;**8**:235.

22. Bjerketorp J, Nilsson M, Ljungh Å, Flock J-I, Jacobsson K, Frykberg L. A novel von Willebrand factor binding protein expressed by Staphylococcus aureus. *Microbiology (Reading, Engl )* 2002;**148**:2037–2044.

23. Viljoen A, Viela F, Mathelié-Guinlet M, Missiakas D, Pietrocola G, Speziale P, Dufrêne YF. Staphylococcus aureus vWF-binding protein triggers a strong interaction between clumping factor A and host vWF. *Commun Biol* 2021;**4**:453.

24. Kroh HK, Panizzi P, Bock PE. Von Willebrand factor-binding protein is a hysteretic conformational activator of prothrombin. *Proceedings of the National Academy of Sciences of the United States of America* 2009;**106**:7786–7791.

25. Thomas S, Liu W, Arora S, Ganesh V, Ko Y-P, Höök M. The Complex Fibrinogen Interactions of the Staphylococcus aureus Coagulases. *Front Cell Infect Microbiol* 2019;**9**:106.

26. Vanassche T, Kauskot A, Verhaegen J, Peetermans WE, van Ryn J, Schneewind O, Hoylaerts MF, Verhamme P. Fibrin formation by staphylothrombin facilitates Staphylococcus aureus-induced platelet aggregation. *Thromb Haemost* 2012;**107**:1107–1121.

27. Liesenborghs L, Verhamme P, Vanassche T. Staphylococcus aureus, master manipulator of the human hemostatic system. *J Thromb Haemost* 2018;**16**:441–454.

28. McDevitt D, Vaudaux P, Foster TJ. Genetic evidence that bound coagulase of Staphylococcus aureus is not clumping factor. *Infect Immun* 1992;**60**:1514–1523.

29. Shannon O, Flock J-I. Extracellular fibrinogen binding protein, Efb, from Staphylococcus aureus binds to platelets and inhibits platelet aggregation. *Thromb Haemost* 2004;**91**:779–789.

30. Lee LYL, Liang X, Höök M, Brown EL. Identification and characterization of the C3 binding domain of the Staphylococcus aureus extracellular fibrinogen-binding protein (Efb). *J Biol Chem* 2004;**279**:50710–50716.

31. Ko Y-P, Kuipers A, Freitag CM, Jongerius I, Medina E, van Rooijen WJ, Spaan AN, van Kessel KPM, Höök M, Rooijakkers SHM. Phagocytosis escape by a Staphylococcus aureus protein that connects complement and coagulation proteins at the bacterial surface. *PLoS Pathog* 2013;**9**:e1003816.

32. Palma M, Shannon O, Quezada HC, Berg A, Flock JI. Extracellular fibrinogen-binding protein, Efb, from Staphylococcus aureus blocks platelet aggregation due to its binding to the alpha-chain. *J Biol Chem* 2001;**276**:31691–31697.

33. Bokarewa MI, Jin T, Tarkowski A. Staphylococcus aureus: Staphylokinase. *Int J Biochem Cell Biol* 2006;**38**:504–509.

34. Rooijakkers SHM, van Wamel WJB, Ruyken M, van Kessel KPM, van Strijp JAG. Anti-opsonic properties of staphylokinase. *Microbes Infect* 2005;**7**:476–484.

35. Haas CJC de, Veldkamp KE, Peschel A, Weerkamp F, van Wamel WJB, Heezius ECJM, Poppelier MJJG, van Kessel KPM, van Strijp JAG. Chemotaxis inhibitory protein of Staphylococcus aureus, a bacterial antiinflammatory agent. *J Exp Med* 2004;**199**:687–695.

36. Rooijakkers SHM, van Kessel KPM, van Strijp JAG. Staphylococcal innate immune evasion. *Trends in Microbiology* 2005;**13**:596–601.

37. Rooijakkers SHM, Ruyken M, Roos A, Daha MR, Presanis JS, Sim RB, van Wamel WJB, van Kessel KPM, van Strijp JAG. Immune evasion by a staphylococcal complement inhibitor that acts on C3 convertases. *Nat Immunol* 2005;**6**:920–927.

38. Nygaard TK, Pallister KB, Dumont AL, DeWald M, Watkins RL, Pallister EQ, Malone C, Griffith S, Horswill AR, Torres VJ, Voyich JM. Alpha-toxin induces programmed cell death of human T cells, B cells, and monocytes during USA300 infection. *PLoS ONE* 2012;**7**:e36532.

39. Berube B, Wardenburg J. Staphylococcus aureus α-Toxin: Nearly a Century of Intrigue. *Toxin* 2013;**5**:1140–1166.

40. Wang R, Braughton KR, Kretschmer D, Bach T-HL, Queck SY, Li M, Kennedy AD, Dorward DW, Klebanoff SJ, Peschel A, DeLeo FR, Otto M. Identification of novel cytolytic peptides as key virulence determinants for community-associated MRSA. *Nat Med* 2007;**13**:1510–1514.

41. Rautenberg M, Joo H-S, Otto M, Peschel A. Neutrophil responses to staphylococcal pathogens and commensals via the formyl peptide receptor 2 relates to phenol-soluble modulin release and virulence. *FASEB J* 2011;**25**:1254–1263.

42. Surewaard BGJ, Haas CJC de, Vervoort F, Rigby KM, DeLeo FR, Otto M, van Strijp JAG, Nijland R. Staphylococcal alpha-phenol soluble modulins contribute to neutrophil lysis after phagocytosis. *Cell Microbiol* 2013;**15**:1427–1437.

43. Grosz M, Kolter J, Paprotka K, Winkler A-C, Schäfer D, Chatterjee SS, Geiger T, Wolz C, Ohlsen K, Otto M, Rudel T, Sinha B, Fraunholz M. Cytoplasmic replication of Staphylococcus aureus upon phagosomal escape triggered by phenol-soluble modulin α. *Cell Microbiol* 2014;**16**:451–465.

44. Krakauer T, Stiles BG. The staphylococcal enterotoxin (SE) family: SEB and siblings. *Virulence* 2013;**4**:759–773.

45. Dinges MM, Orwin PM, Schlievert PM. Exotoxins of Staphylococcus aureus. *Clin Microbiol Rev* 2000;**13**:16–34.

46. Oscarsson J, Tegmark-Wisell K, Arvidson S. Coordinated and differential control of aureolysin (aur) and serine protease (sspA) transcription in Staphylococcus aureus by sarA, rot and agr (RNAIII). *Int J Med Microbiol* 2006;**296**:365–380.

47. Laarman AJ, Ruyken M, Malone CL, van Strijp JAG, Horswill AR, Rooijakkers SHM. Staphylococcus aureus metalloprotease aureolysin cleaves complement C3 to mediate immune evasion. *J Immunol* 2011;**186**:6445–6453.
